# The comparative effect of exposure to various risk factors on the risk of hyperuricaemia: diet has a weak causal effect

**DOI:** 10.1186/s13075-021-02444-8

**Published:** 2021-03-04

**Authors:** Ruth K. G. Topless, Tanya J. Major, Jose C. Florez, Joel N. Hirschhorn, Murray Cadzow, Nicola Dalbeth, Lisa K. Stamp, Philip L. Wilcox, Richard J. Reynolds, Joanne B. Cole, Tony R. Merriman

**Affiliations:** 1 Department of Biochemistry, University of Otago, Dunedin, New Zealand; 2 Programs in Metabolism and Medical & Population Genetics, Broad Institute of MIT and Harvard, Cambridge, MA USA; 3 Diabetes Unit and Center for Genomic Medicine, Massachusetts General Hospital, Boston, MA USA; 4 Department of Medicine, Harvard Medical School, Boston, MA USA; 5 Division of Endocrinology and Center for Basic and Translational Obesity Research, Boston Children’s Hospital, Boston, MA USA; 6 Department of Genetics, Harvard Medical School, Boston, MA USA; 7 Department of Medicine, Faculty of Medical Sciences, University of Auckland, Auckland, New Zealand; 8 Department of Medicine, University of Otago Christchurch, Christchurch, New Zealand; 9 Department of Mathematics and Statistics, University of Otago, Dunedin, New Zealand; 10 Division of Clinical Immunology and Rheumatology, University of Alabama Birmingham, Birmingham, AL USA

**Keywords:** Hyperuricemia, Gout, Risk factor, Population attributable fraction, Variance, Genetic polymorphism, Diet, Mendelian randomisation

## Abstract

**Background:**

Prevention of hyperuricaemia (HU) is critical to the prevention of gout. Understanding causal relationships and relative contributions of various risk factors to hyperuricemia is therefore important in the prevention of gout. Here, we use attributable fraction to compare the relative contribution of genetic, dietary, urate-lowering therapy (ULT) and other exposures to HU. We use Mendelian randomisation to test for the causality of diet in urate levels.

**Methods:**

Four European-ancestry sample sets, three from the general population (*n* = 419,060) and one of people with gout (*n* = 6781) were derived from the Database of Genotypes and Phenotypes (ARIC, FHS, CARDIA, CHS) and UK Biobank. Dichotomised exposures to diet, genetic risk variants, BMI, alcohol, diuretic treatment, sex and age were used to calculate adjusted population and average attributable fractions (PAF/AAF) for HU (≥0.42 mmol/L [≥7 mg/dL]). Exposure to ULT was also assessed in the gout cohort. Two sample Mendelian randomisation was done in the UK Biobank using dietary pattern-associated genetic variants as exposure and serum urate levels as outcome.

**Results:**

Adherence to dietary recommendations, BMI (< 25 kg/m^2^), and absence of the *SLC2A9 rs12498742* urate-raising allele produced PAFs for HU of 20 to 24%, 59 to 69%, and 57 to 64%, respectively, in the three non-gout cohorts. In the gout cohort, diet, BMI, *SLC2A9 rs12498742* and ULT PAFs for HU were 12%, 49%, 48%, and 63%, respectively. Mendelian randomisation demonstrated weak causal effects of four dietary habits on serum urate levels (e.g. preferentially drinking skim milk increased urate, *β* = 0.047 mmol/L, *P* = 3.78 × 10^−8^). These effects were mediated by BMI, and they were not significant (*P* ≥ 0.06) in multivariable models assessing the BMI-independent effect of diet on urate.

**Conclusions:**

Diet has a relatively minor role in determining serum urate levels and HU. In gout, the use of ULT was the largest attributable fraction tested for HU.

**Supplementary Information:**

The online version contains supplementary material available at 10.1186/s13075-021-02444-8.

## Introduction

Hundreds of genetic variants are associated with serum urate levels [1–3], and observational studies have associated individual dietary factors (e.g. alcohol, sugar-sweetened beverages, coffee, red-meat consumption [4–8]) and overall eating habits [9,10] with urate levels, along with other environmental (e.g. diuretic use [11, 12]) and endogenous factors (e.g. age and sex [13]). Understanding the importance of risk factors and their causal relationship (if any) with HU is critical in developing strategies for the prevention of HU and gout. However, addressing causality is challenging. Observational, longitudinal or migratory studies, and temporal correlations can only be regarded as hypothesis-generating owing to the intractable issue of unmeasured confounding. Any attempts to draw conclusions with respect to causality, including using causal language/inferences, even from an accumulation of studies, mis-represents the evidence [14]. In this context, we note that gout is a multi-stage process beginning with HU, progressing to deposition of monosodium urate crystals and culminating in an innate immune response to crystals [15]. Not all people with HU develop gout [16], so HU and the progression from HU to gout should not be conflated when considering possible causal risk factors.

The gold standard for testing an exposure for a causal role is the randomised clinical trial (RCT). This approach has demonstrated causality for dissolved sugar (sugar-sweetened beverages) in raising urate levels [17–19]. However, for the majority of suspected causal exposures an RCT is not possible. Mendelian randomisation (MR) exploits the natural randomisation of alleles causal for a particular exposure and is analogous to a RCT. Several MR studies have shown a small causal effect of BMI on urate levels (0.0045 to 0.010 mmol/L [0.075–0.17 mg/dL] increase in serum urate per unit increase in genetically determined BMI [20–22]). Dietary preferences have a heritable component and genetic associations have been reported [23–25]. Mendelian randomisation in the UK Biobank, using genetic variants associated with dietary patterns has demonstrated that a ‘healthful’ versus ‘unhealthful’ dietary pattern is not strongly causal for coronary heart disease or type 2 diabetes, despite diet being strongly correlated with these diseases [26].

Using the widely applied approach of decomposing variance, where the sum of multiple risk factors included in a model is constrained to 100%, overall diet contributed ≤0.3% of variance in urate levels, substantially less than the 23.9% explained by inherited common genetic variants [10]. Why so little variance is explained is unclear, but one possible reason is that overall diet, which comprises some foods associated with increased urate and some foods associated with decreased urate, does not play a strong causal role. This possibility is supported by a RCT that reported a small 0.021 mmol/L (0.35 mg/dL) reduction in urate levels in people following the Dietary Approaches to Stop Hypertension (DASH) diet compared to those on a ‘typical’ US diet [27]. Another RCT comparing the Mediterranean diet to a ‘prudent Westernised diet’ reported a small reduction in serum urate levels (0.010 mmol/L [0.17 mg/dL]) over 5 years [28]. Another possible explanation postulated in ref. [9] for the small amount of variance explained is low variability in diet within the US cohorts used in [10]. However, differences in diet between men and women, across age groups, socioeconomic status, ethnicity and BMI strata in the US have been reported [29]—to observe these differences there must be variability in the diet.

Population attributable fraction (PAF) is the proportion of cases for an outcome within a population that can be attributed to a given risk factor, incorporating both the prevalence and the effect size of the exposure [30]. The sum of PAFs for multiple risk factors for a single condition is not constrained to 100%, because an outcome can have multiple risk pathways population-wide. Using the Third National Health and Nutrition Examination Survey PAFs were reported of 44% for being overweight or obese (implying that 44% of HU would be prevented if the entire population had BMI < 25 kg/m^2^) and 9% for non-adherence to a DASH-style diet [9]. Variances explained were 8.3% and 0.1%, respectively [9], indicating the two methods agree as to which exposure has a greater impact.

Our first aim was to use attributable fraction to compare the contributions to HU of various genetic, environmental and endogenous risk factors, including lack of use of urate-lowering therapy. The second aim was to use MR to test for a causal role of diet in determining urate levels.

## Participants and methods

### Participants and data collection

The attributable fraction analysis included four distinct cohorts of European ancestry (Table 1)—cohorts 1 to 3 are population-based and the fourth cohort is comprised entirely of people with gout.

**Table 1:** Characteristics of study cohorts

Risk factors	Cohort 1 (US-based)		Cohort 2 (UK-based)		Cohort 3 (UK-based)		Gout cohort (UK-based)	
	Normouricaemia (***n*** = 11,883)	Hyperuricaemia (***n*** = 2364)	Normouricaemia (***n*** = 52,782)	Hyperuricaemia (***n*** = 4469)	Normouricaemia (***n*** = 317,414)	Hyperuricaemia (***n*** = 30,112)	Normouricaemia (***n*** = 4574)	Hyperuricaemia (***n*** = 2207)
Sex - male	4967 (41.8)	1872 (79.2)	21,493 (40.7)	3848 (86.1)	131,853 (41.5)	25,508 (84.7)	4139 (90.5)	2124 (96.2)
BMI ≥25 kg/m^2^	6320 (53.2)	1993 (84.3)	32,651 (61.9)	4033 (90.2)	205,746 (64.8)	27,189 (90.3)	4154 (90.8)	2046 (92.7)
BMI, mean (SD)	25.9 (4.6)	29.1 (4.8)	26.8 (4.6)	30.2 (4.8)	27.1 (4.6)	30.4 (5.0)	30.7 (5.0)	30.7 (4.8)
*SLC2A9*, *rs12498742* A-allele present	11,148 (93.8)	2285 (96.7)	49,619 (94.0)	4358 (97.5)	298,675 (94.1)	29,336 (97.4)	4473 (97.8)	2171 (98.4)
DASH diet non-adherence	11,782 (99.2)	2352 (99.5)	48,005 (90.9)	4231 (94.7)	^	^	^	^
Healthy eating diet non-adherence	^	^	^	^	284,124 (89.5)	27,509 (91.4)	4113 (89.9)	1996 (90.4)
Alcohol ≥1 drink/week	5843 (49.2)	1268 (53.6)	37,721 (71.5)	3604 (80.6)	222,701 (70.2)	23,895 (79.4)	3802 (83.1)	1916 (86.8)
On diuretic therapy	1203 (10.1)	632 (26.7)	3098 (5.9)	886 (19.8)	22,024 (6.9)	6699 (22.2)	761 (16.6)	352 (15.9)
Age ≥50 years	6884 (57.9)	1643 (69.5)	40,199 (76.2)	3581 (80.1)	245,507 (77.3)	24,336 (80.8)	4256 (93.0)	1874 (84.9)
Age, mean (SD)	51.4 (14.9)	54.5 (12.4)	56.4 (8.1)	57.5 (8.0)	56.7 (8.0)	57.7 (7.9)	60.8 (6.4)	58.3 (7.5)
Not treated with urate-lowering therapy	^	^	^	^	^	^	455 (9.9)	1461 (66.2)

All values represent *N* (%) within each cohort

^Data not applicable or not available

Cohort 1 comprised 14,247 participants of European ancestry from the US population—7342 from the Atherosclerosis Risk in Communities (ARIC) Study, 1314 from the Coronary Artery Risk Development in Young Adults (CARDIA) Study, 2513 from the Cardiovascular Health Study (CHS) and 3078 from the Framingham Heart Study (FHS). These numbers exclude people without serum urate measurements or genome-wide genotypes, along with individuals aged under 18 years, people with kidney disease or gout and those taking urate-lowering therapy. People who answered less than 10% of the food frequency survey, those whose estimated average daily calorie intake was less than 600 kcal/day or greater than 4200 kcal/day and those whose questionnaire answers were deemed unreliable by the study interviewer at recruitment were also excluded. For ARIC, one person from each first degree-related pair was excluded.

Cohort 2, cohort 3 and the gout cohort were sourced from the UK Biobank resource. Subjects of European ancestry and who had urate measures and genotypes available were included in the analysis. Relatives with kinship coefficients > 0.177 were removed, and one person from each relationship was kept, with a preference for keeping gout-affected participants. Those who self-reported having kidney disease were also removed. The gout cohort comprised people who self-reported having gout at visit 0 or were being treated with urate-lowering therapy (*n* = 6781) [31], this case definition has been validated [31, 32]. Cohort 2 consisted of UK Biobank participants who answered a 24-h dietary recall questionnaire during the assessment visit (*n* = 57,251) and cohort 3 consisted of the remaining UK Biobank subjects who answered a reduced food frequency questionnaire (*n* = 347,526). We excluded, from cohort 2, subjects who had energy intakes in excess of 18,000 kJ for females and 20,000 kJ for males based on their 24-h dietary recall data, those who had unreliable dietary data as flagged by the recruiter, or subjects not eating normally due to illness or fasting. No additional exclusions were applied for cohort 3 or the gout cohort. Participants used for the MR analysis were also from the UK Biobank, and the MR cohort has been described previously [26].

Collection of dietary and serum urate data are described in the Supplemental material. Collection of genetic data for the ARIC, FHS, CHS and CARDIA cohorts is described in ref. [10] and for the UK Biobank in [33].

### Data dichotomisation

To calculate a PAF, all exposure and outcome variables must be dichotomous. The outcome for this study was HU, defined as serum urate ≥0.42 mmol/L [≥7 mg/dL] for men and women [34]. For the genetic exposures, HU risk alleles were defined as urate-increasing under a dominant model [35]. Determination of dichotomised dietary exposures is described in the Supplemental material. Alcohol exposure was defined as > 1 drink per week, being overweight/obese as BMI ≥25 kg/m^2^, age was dichotomised as ≥50 versus < 50 years partly in order to capture menopause as a risk factor in women and diuretic use either self-reporting or not self-reporting diuretic intake—these variables were the same as those for which PAR estimates were calculated in the Third National Health and Nutrition Examination Survey in ref. [9] and for age. In the gout cohort, self-reported treatment with urate-lowering therapies, allopurinol (*n* = 4841), probenecid (*n* = 3) and sulphinpyrazone (*n* = 21) (the only three urate-lowering medications for which baseline medication data were available) was a dichotomised exposure variable.

### Statistical analysis

All analyses were performed using R v3.6.1 in RStudio 1.2.5019. For the various exposures, the PAF calculation was (frequency of exposure in cases) × (OR_Exposure_ – 1)/OR_Exposure_) [36]. Odds ratios for the risk of HU for these exposures were calculated in a logistic regression multivariable model including all other environmental and endogenous exposure variables and *SLC2A9* rs12498742 genotype—this variant was chosen for individual focus because of its large effect on serum urate levels [35]. For the percent variance explained analysis (Table 3), effect sizes (β) on serum urate levels for the same exposures were calculated in a linear regression multivariable model including all other environmental and endogenous exposure variables and *SLC2A9* rs12498742 genotype. Average attributable fractions (AAFs), adjusted for all other exposure variables and rs12498742 genotype (Table 2) were calculated using a multivariable model in the R function averageAF [36], described in more detail in Supplemental material. Age was removed as it did not confer risk in the gout cohort. In general, AAFs are lower than PAFs calculated from the same data (as consistently observed here) and it has been proposed that they reflect the most plausible/reliable result across the many different methods of calculating attributable fractions [37].

**Table 2:** Population attributable and average attributable fractions for HU risk exposures

	Cohort 1 (US-based)			Cohort 2 (UK-based)			Cohort 3 (UK-based)			Gout cohort (UK-based)		
	OR (95% CI)	PAF (95% CI)	AAF (95% CI)	OR (95% CI)	PAF (95% CI)	AAF (95% CI)	OR (95% CI)	PAF (95% CI)	AAF (95% CI)	OR (95% CI)	PAF (95% CI)	AAF (95% CI)
Sex - male	6.2 (5.4; 7.0)	64.3 (64.6; 67.8)	29.8 (29.2; 30.3)	8.2 (7.5; 8.9)	75.6 (74.6; 76.5)	30.9 (30.2; 31.6)	7.5 (7.3; 7.8)	73.4 (73.0; 73.8)	30.8 (30.2; 31.5)	4.9 (3.7; 6.6)	76.7 (69.9; 81.7)	30.2 (29.7; 30.6)
BMI ≥25 kg/m^2^	3.4 (3.0; 3.8)	59.2 (55.8; 62.2)	23.5 (23.1; 24.0)	4.2 (3.8; 4.6)	68.6 (66.2; 70.8)	26.2 (25.5; 26.8)	3.8 (3.6; 3.9)	66.4 (65.4; 67.3)	25.8 (25.2; 26.4)	2.1 (1.6; 2.7)	48.8 (36.4; 58.4)	14.2 (14.0; 14.2)
*SLC2A9*, *rs12498742* A-allele present	2.4 (1.8; 3.2)	56.6 (43.8; 66.3)	22.0 (21.6; 22.4)	2.9 (2.4; 3.5)	63.8 (56.2; 70.0)	23.5 (22.9; 0.24)	2.7 (2.5; 2.9)	61.7 (58.8; 64.3)	23.1 (22.6; 23.7)	1.9 (1.2; 3.2)	47.8 (16.2; 67.3)	13.5 (13.3; 13.8)
Diet non-adherence	1.3 (0.6; 2.4)^#^	20.3 (54.6; 58.8)^#^	6.3 (6.2; 6.5) ^#^	1.3 (1.2; 1.5) ^#^	23.6 (12.7; 33.0) ^#^	7.1 (6.9; 7.3) ^#^	1.3 (1.2; 1.4) *	21.4 (8.2; 24.4)*	6.5 (6.3; 6.7)*	1.2 (0.9; 1.4)*	11.8 (−7.3; 27.2)*	2.7 (2.6; 2.7)*
Alcohol ≥1 drink/week	1.1 (1.0; 1.2)	5.2 (−0.1; 10.0)	1.5 (1.4; 1.5)	1.3 (1.2; 1.4)	19.5 (14.2; 24.3)	5.7 (5.6; 5.9)	1.3 (1.3; 1.4)	19.4 (17.5; 21.2)	5.9 (5.7; 6.0)	1.1 (0.9; 1.4)	10.0 (−5.8; 23.1)	2.2 (2.2; 2.3)
On diuretic therapy	4.8 (4.2; 5.5)	21.1 (20.3; 21.9)	8.7 (8.6; 8.9)	4.2 (3.8; 4.6)	15.1 (14.6; 15.5)	4.8 (4.7; 5.0)	4.3 (4.2; 4.5)	17.1 (16.9; 17.3)	5.8 (5.6; 5.9)	1.7 (1.4; 2.0)	6.4 (4.6; 7.9)	1.8 (1.7; 1.8)
Age ≥ 50 years	1.3 (1.1; 1.4)	15.1 (8.7; 20.9)	4.7 (4.6; 4.8)	1.0 (0.9; 1.1)	0.4 (−6.4; 6.6)	0.1 (0.1; 0.1)	1.0 (1.0; 1.0)	0.2 (−2.4; 2.7)	0.1 (0.1; 0.1)	^	^	^
Not treated with urate-lowering therapy	^	^	^	^	^	^	^	^	^	21.8 (18.9; 25.1)	63.2 (62.7; 63.6)	33.3 (32.8; 33.7)

^#^Non-adherence to DASH guidelines; *Non-adherence to the Harvard Healthy Eating Pyramid guidelines; ^Data not applicable or variable is not a risk factor in this group

All analyses are adjusted in a multivariable model for the other risk factors. OR calculations were done in a logistic regression model. For the gout cohort in a model including age, the OR was 0.5 (0.4; 0.6). For *SLC2A9 rs12498742*, PAF and AAF were calculated under a dominant model for the A-allele

**Fig. 1:**
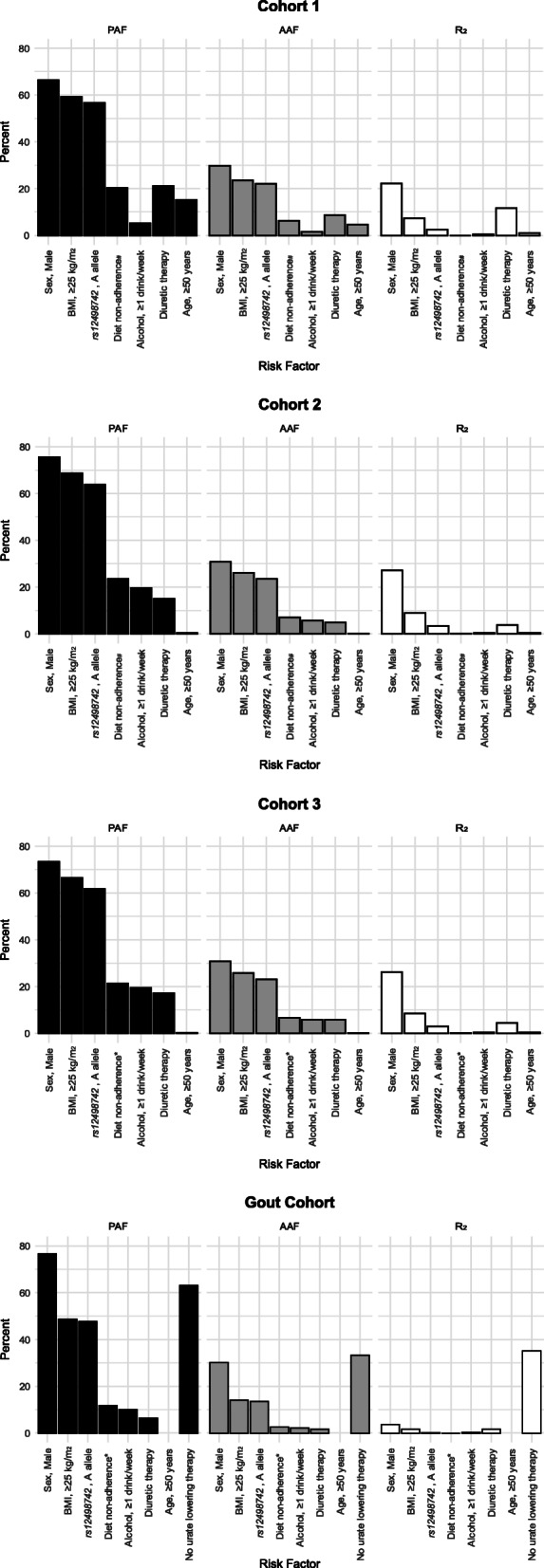
Population attributable fraction, average attributable fraction and variance explained for environmental and endogenous risk exposures for hyperuricaemia or serum urate levels. PAF – population attributable fraction; AAF – average attributable fraction; *R*^2^ – partial *R*^2^ value (*R*^2^_B_) converted to a percentage (*R*^2^ * 100). Population attributable fraction and average attributable fraction values relate to hyperuricemia as a dichotomous variable where for the *SLC2A9 rs12498742* A-allele PAF and AAF was calculated under a dominant model; *R*^2^ relates to serum urate as a continuous variable. All risk factors are dichotomous. All analyses are adjusted in a multivariable model for the other risk factors. ^#^Non-adherence to DASH guidelines; *Non-adherence to the Harvard Healthy Eating Pyramid guidelines. Missing bars in Fig. 1d reflect age not being a risk factor in this group

For genetic variants in Table S2, PAFs (and odds ratios) were calculated per allele under an additive model using the method of Rockhill et al. [36] ((frequency risk allele in cases) × ((OR_RiskAllele_ – 1)/OR_RiskAllele_)). However, PAFs (and odds ratios) in Tables 2, S1 and Fig. 1 were calculated under a dichotomised (dominant) model with the exposed group defined as those with one or more risk allele and were adjusted for dichotomised age, sex, BMI, diet, alcohol and diuretic exposure risks. All AAF analyses used a dominant model for SNP analysis because risk was dichotomised.

### Mendelian randomisation

Two-sample MR using the MendelianRandomization R package [38] was tested for a causal role of dietary habits in determining urate levels. GWAS summary statistics for urate levels in Europeans were obtained from ref. [3] comprising 288,649 individuals; 101 loci with non-ambiguous lead SNPs where no strand assignation issues could have arisen by GWAS which can occur with A/T and G/C variants where the alternative allele is the same as the potential strand-flipped allele. This conservative approach was taken in order to harmonise effect alleles from a meta-analysis of multiple studies using multiple imputation panels. In order to test that this QC did not influence the results, we conducted a sensitivity analysis and repeated the primary Mendelian randomisation with the inclusion of all ambiguous SNPs (Figure S1)—the results were in high concordance. GWAS data for dietary habits were obtained from a study of 455,146 individuals of European ancestry from the UK Biobank [26]. The latter were derived from consumption patterns for 47 single foods asked about in the reduced food frequency questionnaire or 40 principal component-derived dietary patterns with ≥3 non-ambiguous genome-wide significant index SNPs reported in ref. [26] that were also present in the serum urate GWAS summary statistics [3]. Independence of loci was based on the distance-based pruning approach within the source GWAS [3, 26]—loci had to be > 500 kb apart and if there were two in a 1 Mb interval independence was established by plotting and visualisation of inter-marker linkage disequilibrium. The MR was performed using three methods available in the MendelianRandomization R package—inverse-variance-weighted meta-analysis [39], MR Egger (enables detection of pleiotropy [40]) and weighted median (robust to pleiotropy [41]). Significance thresholds were set at *P* < 0.05/87 (5.8 × 10^− 4^) for the inverse-variance-weighted analysis, *P* < 0.05 for the weighted median analysis and intercept *P* > 0.05 for the MR Egger analysis. Our strategy was to identify causal effects from the inverse-variance-weighted analysis corrected for multiple testing followed by sensitivity analysis by weighted median and MR Egger to test the robustness of the results. All three significance thresholds had to be met for a dietary habit to be considered causal. Multivariable MR was conducted to investigate the possible upstream impact of BMI as a common causal link between dietary patterns and serum urate levels using the likelihood-based method on summary data as described by Burgess and Thompson [42], as implemented in the MendelianRandomization R package. This technique uses pleiotropic genetic variants to estimate the direct effect of multiple exposures on an outcome (e.g. BMI and diet on serum urate), with causal estimates representing the independent causal effect of each exposure on the outcome, not operating through the other exposure included in the analysis.

## Results

### Population and average attributable fractions

Male sex had the largest PAF and AAF (64.3 to 75.6%, and 29.8 to 30.9%, respectively, in cohorts 1 to 3), being overweight or obese had the second largest measures (PAF = 59.2 to 68.6%, AAF = 23.5 to 26.2%), and inheriting the *SLC2A9* rs12498742 A-allele had the third largest effect (PAF = 56.6 to 63.8%, AAF = 22.0 to 23.5%). All other factors had PAFs < 25% and AAFs < 10% (Table 2). Cohorts 2 and 3 had the same ranking of risk factors (sex > BMI > rs12498742 > diet adherence > alcohol consumption > diuretic therapy > age), while cohort 1 ranked the same three risk factors first (sex > BMI > rs12498742), before differing in rankings of the remaining four risk factors (diuretic therapy > diet adherence > age > alcohol consumption) (Fig. 1).

In the gout cohort, lack of treatment with urate-lowering therapy had the second largest PAF of 63.2%, after sex (76.7%) (Table 2). The PAFs for BMI and *SLC2A9* rs12498742 were lower than for the non-gout cohorts (57 to 69% in non-gout and 48 to 49% in gout), and non-adherence to the Healthy Eating Pyramid guidelines was 21.4% in non-gout (cohort 3) and 11.8% in gout. Average attributable fraction values showed a similar trend (Fig. 1).

In sex-stratified analysis in the non-gout cohorts, PAFs for rs12498742 were 77.7 to 96.4% in women compared to 49.2 to 58.9% in men (Table S1). Alcohol was not a risk factor in women across all cohorts, nor age in men in cohorts 2 and 3, and gout (Table S1; 95% CI encompassed 1.0). The lower age limit for recruitment into the UK Biobank, which these two cohorts were derived from, was 40 years, which may have influenced the calculation.

In the non-gout cohorts, of 30 genetically-independent serum urate-associated genetic variants chosen as having the top effects by GWAS [33] evaluated (Table S2), the *SLC2A9* rs12498742 variant was the largest, with PAFs ranging from 28.5 to 32.1% and AAFs from 22.0 to 23.5%. For comparative purposes, we summed the PAFs for genetic variants, assuming that the variants act independently of each other to influence the risk of HU, with the individual PAFs summing to > 141% (cohort 1 was 146.2%, cohort 2 was 143.7%, cohort 3 was 141.3%). Summing the AAFs (equivalent to the summing of PAFs, above) resulted in all three cohorts having a summed AAF over 87% (cohort 1 was 97.9%, cohort 2 was 87.1%, cohort 3 was 101.6%). Summed attributable fractions for genetic variants for HU were considerably lower for the gout cohort (PAF was 77.6% and AAF was 44.0%), possibly reflecting selection (collider) bias.

### Percent variance explained for serum urate levels

In the non-gout cohorts, sex had the most percent variance explained (22 to 27%) (Table 3). The dichotomised BMI exposure was consistently 7 to 9%, with diuretic exposure accounting for 12% variance in the US-based cohort and 4–5% in the UK-derived cohorts. The diet estimate was ≤0.1% and *SLC2A9* was 2–3%, similar to our previous report [10]. The gout cohort included urate-lowering therapy exposure in the model, with exposure accounting for the largest proportion (35%) of variance, approximately 10-fold more than any other variable. The use of percent variance explained produced a broadly similar ranking order of risk factors to the PAF and AAF analyses across all four cohorts (Fig. 1).

**Table 3:** Associations of dichotomised risk variables with serum urate levels (including percent variance explained)

Risk factor	Cohort 1 (US-based)		Cohort 2 (UK-based)		Cohort 3 (UK-based)		Gout cohort (UK-based)	
	Beta (95% CI)	***R***^**2**^ (%)	Beta (95% CI)	***R***^**2**^ (%)	Beta (95% CI)	***R***^**2**^ (%)	Beta (95% CI)	***R***^**2**^ (%)
Sex - male	0.078 (0.076; 0.080)	22.4	0.076 (0.075; 0.077)	27.2	0.075 (0.075; 0.076)	25.9	0.062 (0.055; 0.070)	3.7
BMI ≥25 kg/m^2^	0.041 (0.038; 0.043)	7.3	0.039 (0.038; 0.041)	9.0	0.040 (0.039; 0.040)	8.5	0.032 (0.025; 0.039)	1.8
*SLC2A9*, *rs12498742* A-allele present	0.044 (0.039; 0.049)	2.5	0.048 (0.046; 0.050)	3.4	0.047 (0.046; 0.047)	3.0	0.026 (0.009; 0.043)	0.02
Diet non-adherence	0.008 (−0.006; 0.021)^#^	0.01	0.008 (0.006; 0.010)^#^	0.1	0.005 (0.004; 0.005)^*^	0.1	0.004 (−0.003; 0.010)^*^	0.01
Alcohol ≥1 drink/week	0.004 (0.002; 0.007)	0.2	0.008 (0.007; 0.009)	0.4	0.009 (0.008; 0.009)	0.5	0.005 (−0.001; 0.010)	0.3
On diuretic therapy	0.058 (0.054; 0.061)	11.7	0.047 (0.045; 0.049)	3.7	0.049 (0.048; 0.050)	4.5	0.029 (0.029; 0.035)	1.7
Age ≥ 50 years	0.014 (0.012; 0.017)	0.9	0.009 (0.008; 0.010)	0.4	0.009 (0.009; 0.010)	0.4	^	^
Not treated with urate-lowering therapy	^	^	^	^	^	^	0.133 (0.129; 0.138)	35.2

^#^Non-adherence to DASH guidelines; *Non-adherence to the Harvard Healthy Eating Pyramid guidelines; ^Data not applicable or variable is not a risk factor in this group

Beta values represent urate change in mmol/L between risk groups. *R*^2^ – partial *R*^2^ value (*R*^2^_B_) converted to a percentage (*R*^2^ * 100). All analyses are adjusted in a multivariable linear regression model for the other risk factors. Risk for *SLC2A9 rs12498742* was calculated under a dominant model. For the Gout cohort in a model including age, the beta was −0.036 (−0.043; −0.029) and the *R*^2^ was 1.7

### Mendelian randomisation

Five of the 87 single foods and principal component-derived dietary-associated habits [26] provided evidence of a causal effect (IVW *P* < 0.05/87 (5.7 × 10^− 4^)) on urate levels by inverse-variance-weighted MR (Table S3). All five of these dietary habits also had no evidence for an intercept significantly different from zero in the MR Egger analysis (all *P* > 0.05) indicating no evidence for directional (horizontal) pleiotropy. Four of these dietary habits provided evidence for a causal role (*P* < 0.05) and yielded similar effect sizes in the weighted median analysis (Table 4). Two of these causal effects were with dairy-related dietary habits (preferentially drinking skim milk and preferentially drinking milk with a higher fat content), and the other two causal effects were for consuming tub margarine and daily dried fruit consumption.

**Table 4:** Causal effects of dietary habits on serum urate: significant Mendelian randomisation results only

Dietary pattern description	SNP number	Inverse-variance-weighted MR			MR Egger		Weighted median MR	
		β (mmol/L) (95% CI)	***P***_**IVW**_	***P***_**Het**_	Intercept (mmol/L)	***P***_**Intercept**_	β (mmol/L) (95% CI)	***P***_**WM**_
Preferentially drinking skim milk (vs. any other milk type)	3	0.050 (0.032; 0.068)	3.8 × 10^− 08^	9.5 × 10^− 03^	−1.6 × 10^−3^	0.26	0.051 (0.026; 0.075)	3.8 × 10^− 05^
Consuming tub margarine (vs. no spread use)	3	−0.025 (− 0.034; − 0.015)	1.3 × 10^− 07^	3.1 × 10^− 05^	−2.4 × 10^− 3^	0.15	− 0.017 (− 0.032; − 0.001)	0.039
Preferentially drinking milk with a higher fat content*	8	−0.044 (− 0.063; − 0.024)	1.5 × 10^− 05^	5.8 × 10^− 03^	1.2 × 10^−4^	0.87	−0.053 (− 0.070; − 0.036)	2.1 × 10^− 09^
Dried fruit (pieces per day)	25	−0.018 (− 0.028; − 0.008)	3.6 × 10^− 04^	0.001	-6 × 10^−5^	0.77	−0.024 (− 0.035; − 0.013)	3.1 × 10^− 05^

SNP number indicates the number of variants included in the instrumental variable

*P*_Het_ indicates the level of heterogeneity observed between the variants included in the instrumental variable

*Preferred milk type (skimmed vs. semi-skimmed vs. full cream) as a quantitative variable

**Fig. 2:**
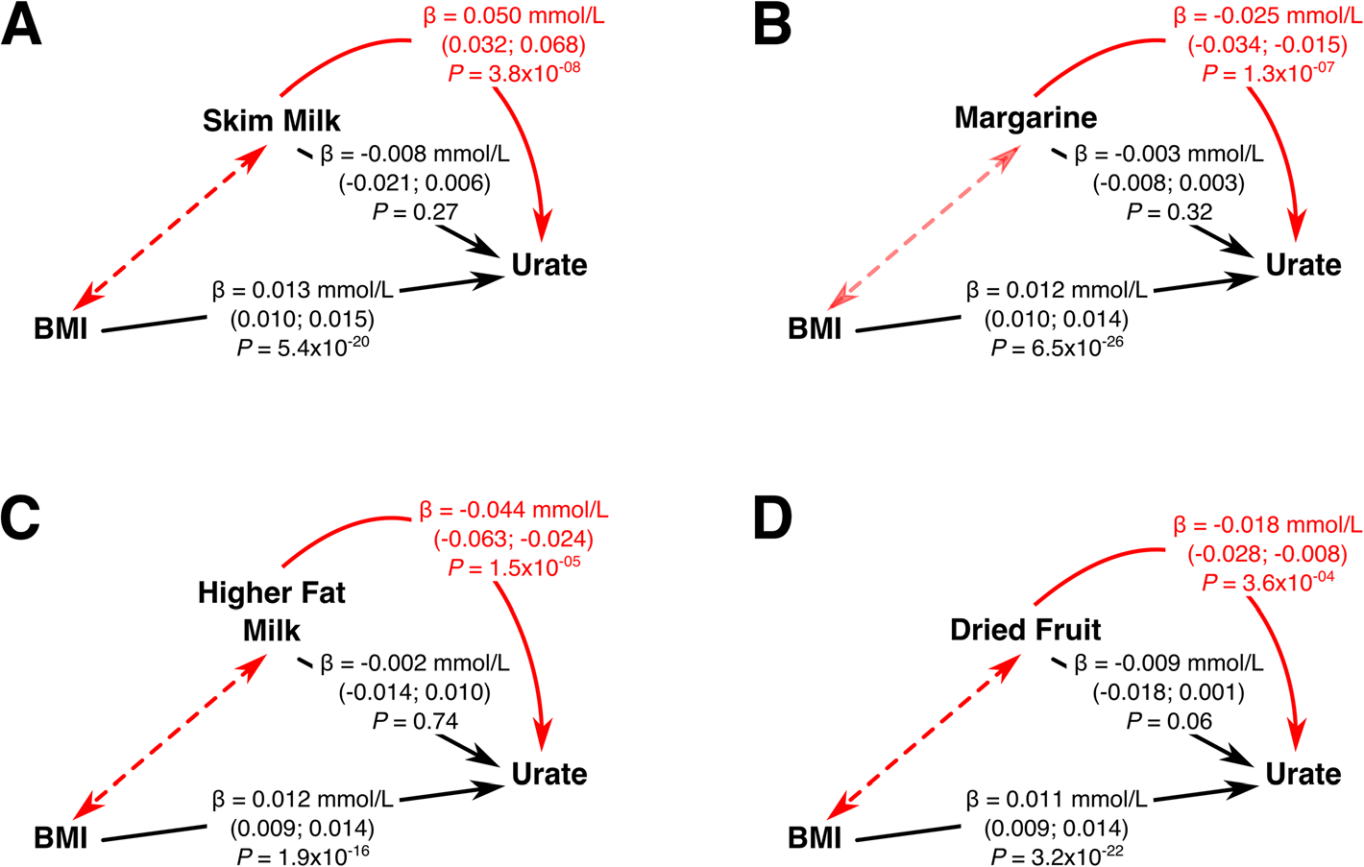
Direct effect of dietary habits on serum urate levels, independent of BMI: multivariable Mendelian randomisation. The solid red arrow and values indicate the causal effect identified in the original inverse variance weighted MR; the dashed red arrow indicates the correlated pleiotropy between BMI and the dietary habit, influencing this original inverse variance weighted MR result; the black arrows and values indicate the causal effect independent of the other exposure variable. Beta values are in mmol/L. Figure 2a relates to results for preferentially drinking skim milk (vs. any other milk type); Fig. 2b relates to results for consuming tub margarine (vs. no spread use)—the dashed red arrow is paler in this figure due to the lower confidence surrounding the correlated pleiotropy; Fig. 2c relates to results for preferentially drinking milk with a higher fat content; and Fig. 2d relates to results for dried fruit consumption (pieces per day)

Of the 39 genetic variants that comprised the four dietary-associated habits, 21 are associated with metabolic traits (http://www.type2diabetesgenetics.org/ [accessed: 2nd June 2020]) and/or traits available in the UKBiobank PheWeb (http://pheweb.sph.umich.edu:5000 [accessed: June 2, 2020]), including 16 specifically associated with BMI or a related body fat trait (Table S4). To test the possibility that the causal association between these four dietary habits and urate levels is due to BMI as a common upstream cause (e.g. change in dietary habits due to weight-loss advice), we applied multivariable MR using the same individual level UK Biobank dataset described in ref. [26]. For all four dietary patterns, including BMI in the multivariable analysis resulted in no evidence for a causal effect (*P* ≥ 0.06), BMI showing a causal relationship with urate levels independent of the dietary habit (Fig. 2). Bidirectional MR between BMI and each of the four dietary habits where, by inverse variance-weighted meta-analysis MR BMI was tested for a causal effect on the dietary habits and each of the dietary habits was tested for a causal effect on BMI, conducted to confirm whether BMI is a common upstream cause of dietary habits, provided evidence in both directions (*P* ≤ 4.4 × 10^− 18^ for BMI to dietary habit, *P* ≤ 9.4 × 10^− 4^ for dietary habit to BMI)—a situation termed “correlated pleiotropy” [43]—except in the margarine analysis for the BMI to dietary habit analysis (*P* = 0.24) although there was evidence for the dietary habit to BMI analysis (*P* = 8.4 × 10^− 61^). This indicates that BMI and the four dietary habits are strongly correlated traits or work through a shared pathway and that the four dietary habits have no effect on urate levels independent of BMI.

## Discussion

Our previous study [10] concluded, using percent variance explained, that common genetic variants have a greater contribution to urate levels in the non-gout population than overall diet. Using attributable fraction measures, we arrive at the same conclusion, importantly also in a cohort of people with gout. Previously, the summed percent variance for the 30 genetic variants for urate levels was 8.7%, considerably greater than the variance explained by the DASH diet [10]. Here, the summed PAFs for the 30 genetic variants was 141 to 146%, considerably greater than that for following the DASH diet recommendations in cohorts 1 and 2. Thus, empirically for HU at least, the different approaches of decomposition of variance and use of attributable fractions provide similar support for the greater relative role of common inherited genetic variation than overall diet in determining urate levels and HU. In the gout cohort, the attributable fractions for urate-lowering therapy were greater than for diet and BMI < 25 kg/m^2^*.* Acknowledging the limitation that we were unable to build compliance, medication dose, and dosing to target into our models (which would contribute to under-estimating the effect of urate-lowering therapy), our data emphasise the importance of gold-standard clinical practice (urate-lowering therapy), to manage HU in gout patients. While weight reduction has established benefits, including to co-morbidities in gout, our data demonstrate the greater impact of urate-lowering therapy in managing HU in gout.

While it is debatable whether public health efforts should be directed to primary prevention of HU, given the lack of evidence that HU is directly causal of conditions other than gout [44], there are two considerations that can be drawn. One, efforts would need to focus on interventions for which there is unequivocal evidence for a substantial impact to be made. This is not the case for the DASH diet (our AAF estimate in a multivariable model of the proportion of cases of HU prevented by following a DASH diet was only 6 to 7%). Two, the proportion of cases of HU attributable to being overweight or obese from the population was 24 to 26% in the same model, only slightly more than the proportion attributable to *SLC2A9* rs12498742 (22 to 24%). It may seem incongruous to compare these exposures in the context of possible public health approaches to prevent primary HU, given that it is not possible to prevent exposure to a common genetic variant. It is, however, possible to modify the impact of a genetic variant. The uricosuric drugs benzbromarone and probenecid inhibit the reuptake of filtered urate by GLUT9 (encoded by *SLC2A9*) [45]; thus, it is conceptually possible to target individuals with the rs12498742 urate-raising allele to improve excretion of urate and prevent HU. From a public health perspective, this is likely a more tractable intervention (in that it targets a single measurable exposure) than preventing obesity, which is caused by multiple environmental and genetic exposures that are not well understood.

That individual foods and estimates of dietary habits associate strongly with urate levels in observational data [10] does not necessarily translate into a clinically significant causal effect. It is interesting to compare association data of the DASH diet score [10] with data from a RCT of the effect of the DASH diet on serum urate levels [27]—the association data show a decrease of 0.023 mmol/L [0.38 mg/dL] between the least and most DASH-like diets in the US population [10], very similar to the 0.021 mmol/L [0.35 mg/dL] decrease when comparing the DASH diet with an ‘average American diet’ in the RCT [27]. In both cases, this is a relatively small change attributable to dietary habits and is reflective of the evidence presented here for a weak BMI-mediated causal relationship between diet and urate levels.

An incongruity is the apparent inconsistency between the two dairy-related MR analyses and results from RCTs [46–48]. Using MR as a complementary approach to investigate causality, we found only a small number of weak causal associations between dietary habits and serum urate. Interestingly, two of the significant causal associations represent opposing dietary habits, namely preferentially drinking skim milk or preferentially drinking milk with a higher fat content. The causal effects observed were consistent with these being opposing dietary habits, with skim milk consumption associating with increased urate, whilst consumption of higher-fat milk associated with decreased urate at an approximately equivalent effect size (0.050 mmol/L [0.84 mg/dL] vs. −0.044 mmol/L [− 0.73 mg/dL], respectively). However, whilst these results are consistent with each other they are not consistent with prior studies of milk and dairy proteins in relation to urate levels. Observational studies have reported an inverse relationship between consumption of dairy products and serum urate levels [7, 10, 49–52]. Many of these observational studies do not separate dairy products into low and high fat content; however, those that do have found that this effect appears to be limited to consumption of skim or low-fat dairy products [10, 51]. RCTs have supported these observational findings [46–48], in particular consumption of skim milk products acutely lowered serum urate levels by approximately 10% in 16 healthy adult men [46]. The apparent inconsistency between the MR and RCT results can be explained by the influence of BMI on the MR analysis. BMI appears to be a common upstream cause in the two dairy-related MR associations reported here, and these two dairy-related dietary habits are highly correlated with BMI, several measures of body fat and weight-loss related traits, including making major dietary changes to lose weight [26] (Table S4). It is plausible that the MR results reflect dietary recommendations given to individuals with a higher BMI (drink skim or low-fat milk), explaining the contradictory results seen here.

Our BMI genetic instrument explains more variance in type of milk consumed (~ 0.5%), than the milk type instruments do themselves (0.04 to 0.1%) [26], highlighting an important limitation to these analyses. Genetic instruments for dietary habits likely explain a small fraction of phenotypic variance [26] or may be linked to diet through indirect mechanisms, potentially subjecting the MR analysis to bias towards the null, pleiotropy or confounding. While multiple MR approaches were used to address some of these pitfalls, future investigation using more biologically based genetic instruments for diet may illuminate previously undetectable causal relationships.

In the sample sets of European ancestry studied here, *SLC2A9* rs12498742 had a considerably greater PAF than the *ABCG2* rs2231142 variant (29 to 32% vs. 6%, respectively (Table S2)). This is because of the 1.7-fold increased effect size of rs12498742 on serum urate levels and the increased prevalence of the urate-increasing allele (77% vs. 11%) [35]. In contrast, in a Japanese study, the PAF for rs2231142 was 29%, compared to 19% for being overweight or obese [53], suggesting that for any primary prevention of HU in the Japanese population, targeting ABCG2 dysfunction would be a strategy to be considered. The rs2231142 risk allele frequency is 29% in the East Asian population compared to 9% in the European population. The authors of the Japanese study concluded that *ABCG2*, at least, is a stronger risk factor for HU than other ‘typical’ environmental risk factors [53].

One limitation of the gout cohort analysis is the possibility of selection (collider) bias resulting from conditioning the sample set on gout ascertainment which would serve, when testing variables that are risk factors for gout per se, to bias effect sizes towards the null or even in an opposing direction [54]. This phenomenon likely explains the reduced (reversed for age) effect sizes for age, sex, BMI and diuretic exposure for each of risk of HU and change in serum urate levels and reduced variance explained, evidenced by non-overlapping 95% CIs compared to cohorts 1 to 3 (Tables 2 and 3). For *SLC2A9*, effect sizes and variance explained were lower in the gout cohort, but some confidence intervals were overlapping. However, for diet and alcohol, there was no difference in effect sizes (the 95% CIs overlapped) suggesting that collider bias does not have a substantive effect on these estimates within the gout cohort. We note that the prevalence of healthy eating diet non-adherence was very similar between the UK Biobank Gout cohort and the equivalent non-gout cohort (cohort 3) indicating that diagnosis of gout did not change dietary behaviour. Selection bias will not influence our effect estimates for urate-lowering therapy; however, estimates for this exposure are likely inflated in the UK Biobank owing to healthy volunteer selection bias [55]. This likely leads to an over-estimate of effect size owing to exposure to urate-lowering therapy, because of a more compliant demographic. Our estimate of OR = 20.2 (Table 2) is considerably higher than a hazard ratio of 4.5 reported for achieving target urate in a gout cohort drawn from the UK primary care population [56]. While our estimate is not representative of the general population, it does indicate the possibility that the relative effect of urate-lowering therapy on HU and serum urate levels is higher when compliance to urate-lowering therapy is increased.

## Conclusions

In conclusion, we demonstrate using attributable fraction measures, that incorporate both the prevalence of exposure and effect size, the considerably greater attributable fraction of HU in the general population owing to common inherited genetic variants and BMI than to dietary exposure. The use of urate-lowering therapy in gout was the largest contributor to attributable fraction of HU. These findings are consistent with previous findings from the use of variance explained in the general population [10]. There is a weak causal effect between four dietary habits and urate levels, all mediated by BMI. Collectively, our findings refute the widely held perception that HU is primarily caused by diet [57–60].

## Supplementary Information

**Additional file 1: Table S1.** Sex-stratified population attributable and average attributable fractions for environmental and endogenous risk exposures for hyperuricaemia. **Table S2.** Population attributable and average attributable fractions for 30 genetic risk exposures for hyperuricaemia. **Table S3.** Causal effects of dietary habits on urate levels: Mendelian randomisation results. **Table S4.** Genetic variants comprising the four dietary habit instrumental variables.

**Additional file 2: Text S1.** Edited averageAF.R script for average attributable fraction confidence interval calculation. **Figure S1.** FISH plot of primary Mendelian randomisation analysis vs analysis including all ambiguous SNPs. Points in the upper right quadrant represent those with consistent effect directions and those in the lower left quadrant represent those with inconsistent effect directions. Nearly all of the points, especially the most significant points, are on the 45 degree line in the upper right quadrant.

## Data Availability

All data used were publicly available. Derivative datasets generated during the current study are available from the corresponding author on reasonable request.

## Abbreviations

AAF: Average attributable fraction
ABCG2: ATP-binding cassette subfamily G member 2
ARIC: Atherosclerosis risk in communities
BMI: Body mass index
CARDIA: Coronary artery risk development in young adults
CHS: Cardiovascular heart study
DASH: Dietary approaches to stop hypertension
FHS: Framingham heart study
GLUT9: Glucose transporter 9
GWAS: Genome-wide association study
HU: Hyperuricemia
IVW: Inverse variance weighted
MR: Mendelian randomisation
PAF: Population attributable fraction
RCT: Randomised clinical trial
SLC2A9: Solute carrier family 2, member 9
ULT: Urate-lowering therapy
