# Supplementary material for: The comparative effect of exposure to various risk factors on the risk of hyperuricaemia: diet has a weak causal effect
Source: Arthritis Res Ther. 2021 Mar 4;23:75. doi: 10.1186/s13075-021-02444-8 (PMC7931603; doi:10.1186/s13075-021-02444-8)
Supplement: Supplementary file 2 — Additional file 2: Text S1. Edited averageAF.R script for average attributable fraction confidence interval calculation. Figure S1. FISH plot of primary Mendelian randomisation analysis vs analysis including all ambiguous SNPs. Points in the upper right quadrant represent those with consistent effect directions and those in the lower left quadrant represent those with inconsistent effect directions. Nearly all of the points, especially the most significant points, are on the 45 degree line in the upper right quadrant. [file 13075_2021_2444_MOESM2_ESM.docx]

**SUPPLEMENTAL MATERIAL**

**The comparative effect of exposure to various risk factors on the risk of hyperuricaemia: diet has a weak causal effect**

Ruth KG Topless^1^*, Tanya J Major^1^*, Jose C Florez^2-4^, Joel N Hirschhorn^2,5,6^, Murray Cadzow^1^, Nicola Dalbeth^7^, Lisa K Stamp^8^, Philip L Wilcox^9^, Richard J Reynolds^10^, Joanne B Cole^2,3,5#^, Tony R Merriman^1#^^

^1^ Department of Biochemistry, University of Otago, Dunedin, New Zealand

^2^ Programs in Metabolism and Medical & Population Genetics, Broad Institute of MIT and Harvard, Cambridge, Massachusetts, USA

^3^ Diabetes Unit and Center for Genomic Medicine, Massachusetts General Hospital, Boston, Massachusetts, USA

^4^ Department of Medicine, Harvard Medical School, Boston, Massachusetts, USA

^5^ Division of Endocrinology and Center for Basic and Translational Obesity Research, Boston Children’s Hospital, Boston, Massachusetts, USA

^6^ Department of Genetics, Harvard Medical School, Boston, Massachusetts, USA

^7^ Department of Medicine, Faculty of Medical Sciences, University of Auckland, Auckland, New Zealand

^8^ Department of Medicine, University of Otago Christchurch, Christchurch, New Zealand

^9^ Department of Mathematics and Statistics, University of Otago, Dunedin, New Zealand

^10^ Division of Clinical Immunology and Rheumatology, University of Alabama Birmingham, Birmingham, Alabama, USA

*These authors contributed equally

^#^These authors contributed equally

^Corresponding author

**Participants and methods**

*Data collection*

For each of the studies that made up Cohort 1 information from the baseline visit was used. Serum urate levels were measured as described in ref (1). Information on diuretic intake was obtained using self-reported medication use. All dietary data (including alcohol consumption) for Cohort 1 was collected using food frequency questionnaires. Participants in the ARIC, FHS, and CHS studies completed similar questionnaires in which they were asked to answer the question “How often, on average, in the past year did you eat [this food]?” by choosing from various frequency categories (66 questions and nine frequency categories for ARIC, 99 questions and six frequency categories for CHS, and 126 questions and nine frequency categories for FHS). Participants in CARDIA answered a validated bespoke diet history of 100 food items using a series of questions, “Do you eat [this food]?” if yes “How much do you usually have?” and “How often do you usually have it?”. Dietary data from the four cohorts were converted to equivalent food items, and the genetic data were obtained, as previously described (1).

For Cohorts 2, 3, and the Gout Cohort, information from the UK Biobank visit 0 was used. Serum urate levels were measured by uricase PAP analysis on a Beckman Coulter AU5800 (https://www.ukbiobank.ac.uk/). Information on use of diuretics or urate-lowering therapy was obtained through a verbal interview on prescription medications conducted by a trained nurse. For Cohort 2 diet information was collected using an online tool relating to food intake “yesterday” (the previous 24 hours). Participants were asked “Did you eat [food category] yesterday?” and if they answered yes, they were asked to provide an estimate of the number of servings eaten yesterday for each food listed within the food category. The questionnaire comprised 10 food categories, with a total of 261 food items across these categories (2). Dietary information for Cohort 3 and the Gout Cohort was derived from a reduced food frequency questionnaire focused on intake of a range of common food and drink items. Participants were asked either “how many [servings] of [this food] do you eat per day?” (numeric answers) or “How often do you eat [this food]?” (5 answer categories). A total of 17 food and drink items were included in this questionnaire. For all of the UK Biobank-derived cohorts, alcohol intake was determined from the question “About how often do you drink alcohol?” with response categories “daily or almost daily,” “three or four times a week,” “once or twice a week,” “one to three times a month,” “special occasions only,” and “never.” Cohorts 2, 3, and Gout were genotyped by Affymetrix using the UK BiLEVE Axiom array or the UK Biobank Axiom array and imputed by the UK Biobank using the Haplotype Reference Consortium as the primary imputation reference panel (3).

*Dietary data dichotomisation*

Adherence to the DASH diet defined as either following six or more of the DASH dietary guidelines (using the guides as lower limits for healthy foods e.g. fruit and vegetable intake, and upper limits for unhealthy foods e.g. red meat) or not following the DASH diet guidelines. For Cohort 3 and the Gout Cohort, due to the reduced number of dietary variables available in the UK Biobank food frequency data adherence to the Harvard Healthy Eating Pyramid guidelines (4) was used instead of the DASH diet. The UK Biobank food frequency data did not include intake questions for oils, nuts, legumes, eggs, or dairy products (other than cheese), thus recommendations for these items were unable to be assessed. The Healthy Eating Pyramid guideline recommending moderate alcohol consumption was also not included in this definition as alcohol was included in the analyses separately. Participants were dichotomised based on following six or more of the eight remaining dietary recommendations – taking multivitamins, eating wholegrains three or more times per day, eating vegetables four or more times per day, eating fruit two or more times per day, eating fish or poultry two or less times a day, eating cheese once or twice per day, eating red meat less than once a day, and eating processed grains less than once a day.

In the female-only analysis, menopausal status was dichotomised based on self-report (pre- or post-menopause), those with unknown menopausal status were excluded for these analyses. Menopause risk and associated PAF was not calculated in the female-only Gout Cohort due to 22% of subjects missing menopausal status.

*Statistical analysis*

For AAF the multivariable model in the R function averageAF (5) uses a generalised logistic model for HU and multiple (k) binary covariates, taking covariates off sequentially, then changing the order of sequential removal and running over k! permutations. The AAF is the average (mean) number of predicted cases attributable to each covariate separately over all k! permutations divided by the observed number of cases. The standard error of the mean number of predicted cases was calculated (standard deviation divided by k! permutations) then converted to 95% confidence intervals around the mean. These were subsequently divided by the observed number of cases to create the AAF confidence intervals for each covariate (Text S1). While we calculated PAF a single time, adjusted for all known or available confounding risk factors, AAF is calculated from multiple permutations, each adjusted for a subset of those known or available confounding risk factors (ranging from unadjusted to fully adjusted, with all possible adjusting orders in between) (5,6). This reduces the influence a single confounding risk factor can have on the AAF and recognises that multiple risk factors are involved in disease risk and may work through a shared risk pathway (6). The method also ensures the order of removal of confounding risk factors from the permutations does not unduly influence the result obtained, by averaging the result across all possible confounding risk factor removal orders.

**Table S1.** Sex-stratified population attributable and average attributable fractions for environmental and endogenous risk exposures for hyperuricaemia (Excel file)

**Table S2.** Population and average attributable fractions of serum urate-associated genetic variants for hyperuricemia (Excel file)

**Table S3.** Causal effects of dietary habits on urate levels: Mendelian randomisation results (Excel file)

**Table S4.** Genetic variants comprising the four dietary habit instrumental variables (Excel file)

**Text S1.**  Edited averageAF.R script for average attributable fraction confidence interval calculation, originally sourced from Rückinger *et al.* (5).

**Figure S1 FISH plot of primary Mendelian randomization analysis vs analysis including all ambiguous SNPs.** Points in the upper right quadrant represent those with consistent effect directions and those in the lower left quadrant represent those with inconsistent effect directions. Nearly all of the points, especially the most significant points, are on the 45 degree line in the upper right quadrant.

**Text S1** Edited averageAF.R script for average attributable fraction confidence interval calculation

##############

# averageAF

# Simon Rückinger, Rüdiger von Kries, and André Michael Toschke

# January 2009

# PMID: 19166593

#

# Edited by: Ruth Topless and Tanya Major

# February 2020

#

averageAF = function(formula = NULL, data = NULL) {

d = model.frame(formula, data)

if (any(!(d == 1 |

d == 0)))

stop("only dichotomized (0/1) variables allowed")

vars = ncol(d) - 1

n = nrow(d)

obs.cases = sum(d[, 1])

m = glm(formula, family = binomial, data = d)

if (any(m$coef[-1] < 0))

stop("at least one variable is not a risk factor")

perm = function(from, to, vec) {

if (to == 1)

return(matrix(vec, from, 1))

else if (from == 1)

matrix(vec, 1, to)

else{

X = NULL

for (i in 1:from) {

X = rbind(X, cbind(vec[i], Recall(from - 1, to - 1, vec[-i])))

}

return(X)

}

}

indices = perm(vars, vars, 2:(vars + 1))

pred.cases.m = matrix(NA, nrow = nrow(indices), ncol = vars)

prev.cases.m = matrix(NA, nrow = nrow(indices), ncol = vars)

d1 = d

for (i in 1:nrow(indices)) {

for (k in 1:vars) {

d[, indices[i, k]] = rep(0, n)

pred.cases.m[i, k] = sum(predict.glm(m, d, "response"))

}

d = d1

}

pred.cases.m = cbind(rep(obs.cases, nrow(indices)), pred.cases.m)

for (i in 1:nrow(indices)) {

for (k in 1:vars) {

prev.cases.m[i, indices[i, k] - 1] = pred.cases.m[i, k] - pred.cases.m[i, k + 1]

}

}

prev.cases = apply(prev.cases.m, 2, mean)

PARF = prev.cases / obs.cases

names(PARF) = names(d)[-1]

# return(PARF)

#### added code segment

# original code: calculates average number of predicted cases, then calculates proportion of original cases from that average

# calculate s.e. of mean predicted case number

prev.cases.se = apply(prev.cases.m, 2, sd)

prev.cases.se = prev.cases.se / sqrt(nrow(indices))

# calculate upper/lower CI

t_value <- qt(p = 0.975, df = nrow(indices) - 1)

prev.cases.upper = prev.cases + (t_value * prev.cases.se)

prev.cases.lower = prev.cases - (t_value * prev.cases.se)

# convert to proportion of original cases

SARF_up = prev.cases.upper / obs.cases

names(SARF_up) = names(d)[-1]

SARF_low = prev.cases.lower / obs.cases

names(SARF_low) = names(d)[-1]

# create dataframe of values to return

output = data.frame(

Variable = names(d)[-1],

PARF = PARF,

LowerCI = SARF_low,

UpperCI = SARF_up

)

return(output)

####

}

**References**

1. Major TJ, Topless RK, Dalbeth N, Merriman TR. Evaluation of the diet wide contribution to serum urate levels: meta-analysis of population based cohorts. BMJ 2018;363:k3951.

2. Liu B, Young H, Crowe FL, et al. Development and evaluation of the Oxford WebQ, a low-cost, web-based method for assessment of previous 24 h dietary intakes in large-scale prospective studies. Publ Health Nutr 2011;14:1998-2005.

3. Bycroft C, Freeman C, Petkova D, et al. The UK Biobank resource with deep phenotyping and genomic data. Nature 2018;562:203-209.

4. Department of Nutrition Harvard. The Nutrition Source: Harvard T.H. Chan School of Public Health 2015 [Available from: http://www.hsph.harvard.edu/nutritionsource/healthy-eating-plate/.

5. Rückinger S, von Kries R, Toschke AM. An illustration of and programs estimating attributable fractions in large scale surveys considering multiple risk factors. BMC Med Res Methodol 2009;9:7.

6. Eide GE, Gefeller O. Sequential and average attributable fractions as aids in the selection of preventive strategies. J Clin Epidemiol 1995;48:645-655.
